# The genome of the brown alga *Ectocarpus siliculosus *contains a series of viral DNA pieces, suggesting an ancient association with large dsDNA viruses

**DOI:** 10.1186/1471-2148-8-110

**Published:** 2008-04-12

**Authors:** Nicolas Delaroque, Wilhelm Boland

**Affiliations:** 1 Max-Planck-Institut für Chemische Ökologie, Beutenberg Campus, Hans Knöll Str. 8, D - 07745 Jena, Germany

## Abstract

**Background:**

*Ectocarpus siliculosus *virus-1 (EsV-1) is a lysogenic dsDNA virus belonging to the super family of nucleocytoplasmic large DNA viruses (NCLDV) that infect *Ectocarpus siliculosus*, a marine filamentous brown alga. Previous studies indicated that the viral genome is integrated into the host DNA. In order to find the integration sites of the viral genome, a genomic library from EsV-1-infected algae was screened using labelled EsV-1 DNA. Several fragments were isolated and some of them were sequenced and analyzed in detail.

**Results:**

Analysis revealed that the algal genome is split by a copy of viral sequences that have a high identity to EsV-1 DNA sequences. These fragments are interspersed with DNA repeats, pseudogenes and genes coding for products involved in DNA replication, integration and transposition. Some of these gene products are not encoded by EsV-1 but are present in the genome of other members of the NCLDV family. Further analysis suggests that the *Ectocarpus *algal genome contains traces of the integration of a large dsDNA viral genome; this genome could be the ancestor of the extant NCLDV genomes. Furthermore, several lines of evidence indicate that the EsV-1 genome might have originated in these viral DNA pieces, implying the existence of a complex integration and recombination system. A protein similar to a new class of tyrosine recombinases might be a key enzyme of this system.

**Conclusion:**

Our results support the hypothesis that some dsDNA viruses are monophyletic and evolved principally through genome reduction. Moreover, we hypothesize that phaeoviruses have probably developed an original replication system.

## Background

For several years, the investigation of novel dsDNA viruses has excited the imagination of virologists and evolutionists, prompting the development of several original theories about the evolution of certain dsDNA viruses and their role in shaping the genome of the organisms of the tree of life. It has been proposed that dsDNA viruses were the evolutionary origin of the eukaryotic nucleus or at least that they have contributed to its creation by transferring genetic information to the nucleus [1-7].

The recent discovery and characterization of several large dsDNA viruses from aquatic environments belonging to the phycodnavirus and mimivirus families led to the development of new hypotheses [8-10]. A comparative analysis of the gene content of these viruses with poxviruses, iridoviruses and asfarviruses indicated that they have nine gene products in common, and 33 more gene products are present in at least two of these families. It follows that they might have a common evolutionary ancestor, a nucleocytoplasmic large dsDNA virus (NCLDV) [11,12]. Although most evolutionists seem to accept the hypothesis of a common ancestor, there is no consensus concerning the general morphology of the ancestral NCLDV and how it evolved to give rise to the different classes of viruses. On the one hand, the ancestral NCLDV is believed to have been a dsDNA virus which has evolved by acquiring genes from the host and bacterial endosymbionts and gene duplication [13]. On the other hand, the ancestral NCLDV could have been a huge virus or even a cellular organism that evolved through genome regression [9,14-16].

A well-studied NCLDV is *Ectocarpus siliculosus *virus-1 (EsV-1), a phaeovirus that infects a small marine filamentous alga, *Ectocarpus siliculosus*. Phaeoviruses belong to the Phycodnavirus family. They possess icosahedral morphologies with internal lipid membranes and large double-stranded DNA genomes [17]. EsV-1 and other phaeoviruses only infect free-swimming, wall-less gametes or spores. One copy of the DNA is integrated into the cellular genome and then transmitted via mitosis through all cell generations of the developing host [18-20]. The viral genome remains latent in vegetative cells and is expressed in cells of the reproductive algal organs, sporangia and gametangia only when stimulated by, for example, changes in light composition and temperature [17,21]. Infected algae show no apparent growth or developmental defects other than partial or total inhibition of reproduction and phaeoviruses are pandemic in several brown algal species examined [17]. In addition to its lysogenic life cycle, the genome structure is the major feature which distinguishes EsV-1 from other NCLDVs. The genome is circular, but sequencing the genome resulted in a linear molecule of 335,593 bp ending in inverted repeats. Such a molecule might be able to assemble and form a circular genome [22,23]. It also contains numerous single-stranded regions randomly distributed over the genome whose functions remain unknown [24].

Another characteristic of the genome is its low gene density compared to other NCLDV genomes. The 231 genes occupy only 70 % of EsV-1 genome; these were assembled in islands of densely packed genes that are separated by large regions of DNA repeats and noncoding sequences [23]. In fact, the EsV-1 genome resembles a small eukaryotic chromosome more than a typical viral genome. A comparative analysis of the EsV-1 genome with the genome of two other phycodnaviruses indicated that they could have evolved by loss of genes from a common ancestor, possibly an endosymbiotic organism, thereby supporting the theory of genome regression [25].

To elucidate how the EsV-1 genome is replicated, we first tried to determine where the viral genome is integrated into the host genome. We screened a cosmid library of *Ectocarpus *algae infected by EsV-1 using labelled EsV-1 DNA as a probe. Sequencing and analysis showed that the host chromosomal DNA was interspersed with single copies of small fragments of viral DNA. However, the fragments diverged in their DNA sequence from the corresponding EsV-1 DNA sequences. In the vicinity of these segments, we found other viral genes and pseudo-genes, some of which are present only in the genome of the mimivirus. This finding indicates that the *Ectocarpus *genome contains remnants of the genome of either one giant dsDNA virus or one unicellular organism; this organism could be the ancestor of the contemporary NCLDVs.

Furthermore, this hypothesis is supported by previous data, indicating that the viral integrated fragments might be reassembled to constitute the EsV-1 genome. A putative protein coded by the EsV-1 and algal genome might be a key enzyme of this mechanism due to its similarity to tyrosine recombinases.

## Results

To determine where the viral genome is integrated in the host chromosome, we screened a genomic library of *Ectocarpus siliculosus *sporophyte NZVicZ14 infected by EsV-1. The strain NZVicZ14 was created by mating a male gamete from the strain NZ88 15d2 m containing the EsV-1 genome with a non-infected female gamete from the strain Vic88 12–15 f [26]. The DNA used for constructing the NZVicZ14 library was isolated from filaments prior to the appearance of symptoms and EsV-1 virions in order to limit the number of positive clones containing viral DNA from EsV-1 particles. The library was screened with radio-labeled EsV-1-DNA that had been digested with the DNA restriction enzyme *Sau*3A. The screening isolated 200 positive clones whose cloned fragments have an average size of 35 kb. To distinguish clones containing EsV-1 DNA linked to algal DNA from clones containing only EsV-1 DNA, the isolated positive clones were screened with labelled algal DNA from Vic88 12–15 f female gametophytes.

Surprisingly, all the clones were positive, indicating that they also have algal DNA. Consequently, no clone possessing only EsV-1 DNA was isolated. This result may be due either to the absence of viral particles in the algae or to the single-stranded DNA regions of the EsV-1 genome: these regions make the viral DNA fragile and therefore might render large DNA fragments of EsV-1 in the cosmid vector unclonable [22].

**Figure 1:**
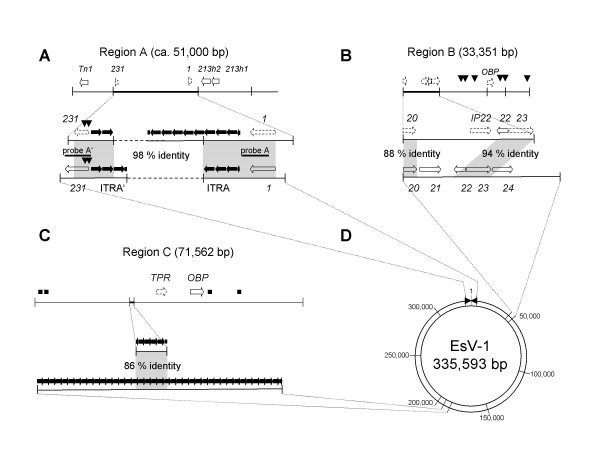
**Genetic organization and comparative genomic analysis of three *Ectocarpus *algal genomic regions**. A, B, and C, maps of the algal genomic and portions of the EsV-1 genome. Gray regions connect homologous genomic regions. Dashed line, unknown DNA sequence. Arrow, putative ORF; broken arrow, pseudo-gene; black arrow, DNA repeats; black square, remnant of ankyrin repeats; arrowhead, relic of transposase; Tn, transposase; numbers indicate ORFs or homologues of the EsV-1 genome; 213h1 and 213h2, homologues of EsV-1 ORF 213; OBP, origin binding protein; IP22, Isoleucine/leucine proline repeat; TPR, tetratricopeptide-repeat. D, Circular map of the EsV-1 genome. Triangles, the inverted terminal repeats, ITRA and ITRA'. Numbers, nucleotide coordinates.

The terminal regions of the EsV-1 genome are thought to be recognized by a putative protelomerase coded by the EsV-1 genome which could in turn linearize the EsV-1 genome and form telomeres [27]. So far, however, experiments to investigate this possibility have not succeeded. To investigate the hypothesis that the terminal repeats might also be the sequences involved in the integration of the viral DNA into the host genome, a second screen was performed using the probes A and A' corresponding to the terminal regions of the viral genome (Fig. 1). Of the 200 positive clones previously isolated, only four overlapping clones hybridized to both probes. Therefore, other clones have viral DNA that is not related to the terminal region. The DNA fragments of the positive clones can be assembled to form a 51-kb genomic region; such a region has been almost entirely sequenced and analyzed (Table 1 and Fig. 1, region A). The genomic region contains a ca. 5430 bp fragment that is 98 % identical to a segment overlapping the terminal region of the EsV-1 genome (Table 1, Fig. 1). The region of identity of the viral fragment and the EsV-1 genome is located just upstream of the promoter region of ORF 1 and ends approximately in the middle of ORF 231 (Fig. 1). The protein product of ORF 231 has no counterpart in the protein data banks [23]. Moreover, a recent blast search of the region indicated that ORF 231 is probably not a gene, since pieces of another ORF situated in ORF 231 code for remnants of a transposase which is related to those coded by the EsV-1 genome (Fig. 1) [23].

**Table 1:** Comparison of the viral integrated fragments of regions A, B, C *vs *the EsV-1 genome.

*Ectocarpus *genomic region	G + C of the genomic region (%)	Position of the viral integrated fragments	G + C of the viral fragments (%)	Position of the similar fragment in the EsV- 1 genome	Identity
A	52.30				
		7036 – 8941	48.22	332959 – 3720	5431/5414 (98 %)
		11748 – 15413	50.8		
B	51.3				
		7 – 934	52.5	37542 – 38469	833/938 (88%)
		6586 – 9708	51.5	40867 – 43989	2978/3153 (94%)
C	53.3				
		25376 – 26459	51.75	190061 – 188934	989/1144 (86%)

The integrated viral fragment contains the same DNA inverted repeats in the same orientation as that which characterizes the EsV-1 genome. Moreover, region A contains additional similar repeats at one extremity (Fig. 1). Apparently, the terminal DNA repeats of EsV-1 genome are longer than previously reported [23] (unpublished results). The discrepancy in the DNA sequence between the proviral and EsV-1 DNA sequences is due to small mutations, deletions or insertions, indicating that the integrated fragments have either accumulated mutations during the evolution or come from an *Ectocarpus *virus other than EsV-1.

**Figure 2:**
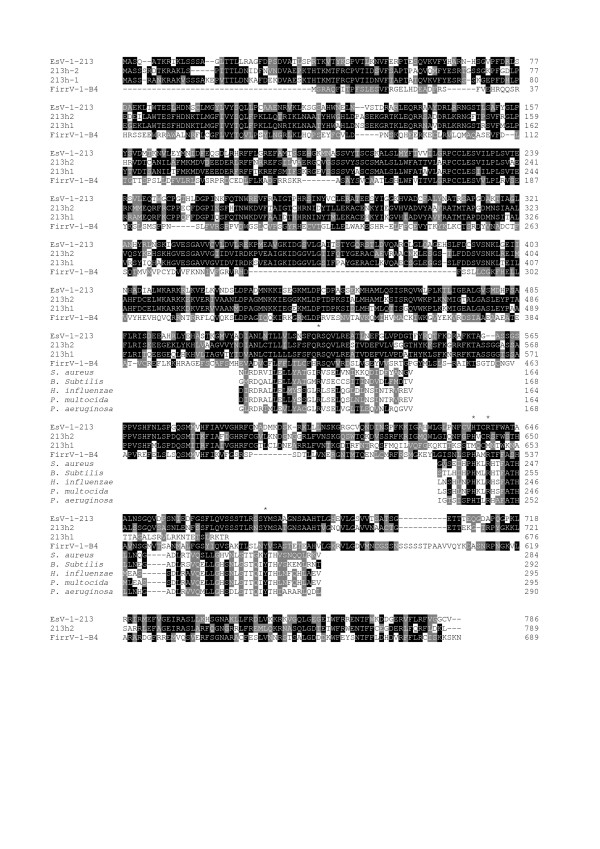
**Alignment of the putative viral integrases of *Ectocarpus *(213h1 and 213h2), EsV-1 and FirrV-1 with segments of tyrosine recombinases from various bacteria**. Accession numbers: AAK14627 (EsV-1), AAR26879 (FirrV-1), Q9KJF6 (*Staphylococcus aureus*), NP_389496 (*Bacillus subtilis *str. 168), P44818 (*Haemophilus influenzae*), AAK03785 (*Pasteurella multocida *str. Pm70) and Q9HXQ6 (*Pseudomonas aeruginosa*). Asteriks indicate the four invariant amino acids of the tyrosine recombinase domain (Arg, His, Arg, Tyr).

Several genes and remnants of genes were found in the vicinity of the viral fragments. Two homologues of ORF 213 of EsV-1 are present in tandem, upstream of ORF 1 (213h1 and 213h2, Fig. 1). ORF 213 is capable of encoding a large enzyme; this enzyme might be involved in DNA integration and recombination (Fig. 2). Protein 213 possesses at its C-terminus a tyrosine-recombinase domain resembling those found in the members of the integrase family whose hallmark is an invariant pattern of four amino acids (Arg, His, Arg, Tyr) forming the catalytic site [28]. ORF 213h1 and 213h2 do not have any similarity in their DNA sequences with ORF 213, indicating that ORF 213h1 and 213h2 are either algal genes or viral genes that NZVic14 horizontally acquired from another virus. The second copy of the gene is truncated at its C terminus resulting in the absence of a part of the tyrosine recombinase domain (Fig. 1 and 2). A homologue of ORF 213 was also found in the genome of another phaeovirus, the *Feldmannia irregularis *virus (FirrV-1), signifying that protein 213 might be essential for the life cycle of the phaeoviruses [25].

A putative transposase, Tn1, which shares identity with eukaryotic transposases of the Fot 1 family, is also present. It is predicted to contain a putative intron with 5'-AG/GUGAGG and 3'UGCAG/GU splice site sequences as well as a branch point UCAC. The genes of EsV-1 genome do not contain introns, whereas these are common in the algal genes. This therefore supports the contention that region A is composed of viral DNA framed by algal DNA.

**Figure 3:**
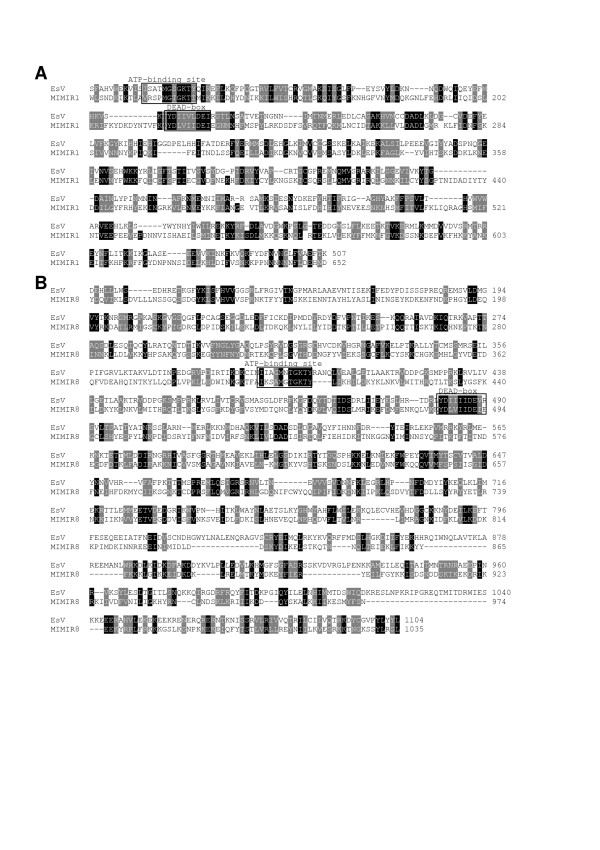
**Alignment of segments of *Ectocarpus *OBPs with the mimiviral counterparts**. A, OBP of region B with MIMIR1 (YP_142355). B; OBP of region C with MIMIR8 (YP_142362). The putative ATP binding site and DEAD region of helicases' superfamily are boxed.

In order to know whether other clones contain viral DNA, several DNA inserts were partially sequenced and analyzed. Viral DNA identical to EsV-1 DNA was missing. Of these sequenced inserts, one contains viral DNA at one extremity (Table 1; Fig. 1, region B). This clone, which has been sequenced to completion and analyzed, contains two fragments which have 88 and 94 % identity to EsV-1 DNA (Fig. 1 and Table 2). The fragments possess pseudo- and homologues of ORF 20, 22 and 23 from EsV-1 (Fig. 1). They are arranged as in the EsV-1 genome but diverge at the DNA level. Moreover, region B has a pseudogene whose translated product contains FNIP repeats. The pseudogene is situated between ORF 20 and ORF 22 instead of ORF 21 (Fig. 1). Interestingly, the EsV-1 genome does not contain FNIP repeats; in contrast, the mimivirus genome codes for at least 8 proteins containing this class of repeat [9,29]. Remarkably, the region contains another pseudogene coding for an origin binding protein (OBP); this OBP has its best identity with the MIMIR1 protein, one of the two putative OBPs of the giant mimivirus (Fig. 3 and Table 2). Although the identity is low, the UL-9 helicase domain with its ATP-binding site and DEAD site is still recognizable (Fig. 3). The OBP is also coded by the genome of the Herpes virus and Asfarvirus but is absent in the EsV-1 genome. OBP protein plays a major role in the replication of viral DNA [30]. In addition, region B contains relics of transposase as does region A (Fig. 1B).

**Table 2:** Putative and incomplete ORFs in the three *Ectocarpus *genomic regions.

ORF	G+C content	Position	Size (aa)	Blast (E value)	Amino acid identity	Homolog(s) [accession number]^a^	Putative fonction/Features/comments^b^
213h1	54.24	21498 – 19471	675	0.0	350/615 (56%)	EsV-1-213 (length 786 aa) [AAK14627]	Truncated homologue of EsV-1-213
				2e-35	138/551 (25%)	FirrV-1-B4 (length 689 aa) [AAR26879]	
213h2	54.46	180467 – 15681	788	0.0	443/787 (56%)	EsV-1-213 (length 786 aa) [AAK14627]	Integrase Phage integrase family (aa 490 – 690) [PF00589]
				1e-73	213/770 (27%)	FirrV-1-B4 (length 689 aa) [AAR26879]	
				0.43	27/98 (27%)	*Bacillus subtilis *site-specific integrase/recombinase B4 (length 304 aa) [NP_389496]	
				0.95	38/85 (45%)	*Staphylococcus aureus *subsp. MRSA252 putative integrase/recombinase (length 298 aa) [YP_040639]	
Tn1	56.95	6788 – 4524	541	1e-32	132/524 (25%)	*Aspergillus nidulans *FGSC A4 putative transposase (length 554 aa) [EAA60308]	Transposase helix-turn-helix, Psq domain (aa 1 – 51) [PF05225] [PS50960],
				7e-32	110/384 (28%)	*Aspergillus awamori *transposase (length 555 aa) [AAC49623]	DDE superfamily endonuclease (aa 160 – 372) [PF03184] Contains a putative intron (bp 515 – 1154)
22h1	54.68	7793 – 6684	369	1e-139	250/289 (86%)	EsV-1-22 [NP_077507] (length 284 aa)	
OBP	47.12	41598 – 45086	1162	6e-11	160/730 (21%)	*Acanthamoeba polyphaga *Mimivirus helicase, similar to origin binding protein (length 1052 aa) [YP_142362]	Origin binding protein AAA (aa 386 – 562) [SM00382] [PF00004], Herpes_ori_bp (aa 401 – 930) [PF02399],
				2e-07	65/275 (23%)	*Acanthamoeba polyphaga *Mimivirus helicase, similar to origin binding protein (length 795 aa) [YP_142355]	ATP/GTP-binding site motif A (P-loop) (aa 394 – 401) [PS00017], Helicase domain (aa 381 – 720) [SM00490] [PF00271] [PS51192] [PS51194].

^a ^Accession numbers are from the GenBank database.

^b ^Pfam, prosite or Smart codes are in brackets.

The presence of an OBP-like gene immediately raises the question whether other mimiviral-like genes are present elsewhere in the NZVicV14 *Ectocarpus *genome. Other DNA inserts were sequenced and analyzed. A genomic region of 70 kb was assembled with fragments of positive clones (region C, Fig. 1C). Region C contains a small viral DNA insert having a few repeats that are almost identical to those present in region D of the EsV-1 genome (Fig. 1 and Table 1). Surprisingly, it also has a complete ORF, which is capable of encoding a large OBP. This putative OBP shares identity with MIMIR8, the second and largest OBP coded by the genome of the mimivirus (Fig. 3). Like its MIMIR8 and herpes viral counterparts, this OBP is made of the N-terminal domain of the archaeo-eukaryotic primase (AEP) fused to the UL-9-like helicase domain containing an ATP-binding site and DEAD box [31]. However, the Zinc domain present in MIMIR8 is absent in *Ectocarpus *OBP. No additional ORF of viral origin was found except vestiges of genes coding for tetratricopeptide-repeats (TPR) and ankyrins (Fig. 1). Both kinds of repeats are the most frequent motif detected in putative ORFs of the mimivirus, while ankyrin-repeats were detected only in the EsV-1 genome [9,23]. Current sequencing of other clones indicates that the *Ectocarpus *genome contains several other genes of EsV-1 that could be assembled to form an apparently complete viral genome.

The variation in GC content in chromosomes is a good indicator of recent transposition and integration occurrences. The GC content, which is close to 52 % for the three genomic regions and the integrated viral fragments, shows no traces of a recent integration event (Table 1). Moreover, the GC content of the genomic regions is identical to that of the EsV-1 genome, which is much more than the 28 % GC content of the mimivirus [23,9]. In addition, the GC content of the OBP-like gene of the region C is 47 %, whereas it is only 28 % for the mimiviral gene (Table 2) [9]. Other reported ORFs have a similar GC content close to 52 % (Table 2). Taken together, these data suggest that the integration event of an EsV-like virus must have occurred a long time ago.

## Discussion

### *Ectocarpus' *genome illustrates the evolution of NCLDVs

In this study, we showed that the genome of *Ectocarpus siliculosus *contains proviral fragments that share a high identity with regions of the genome of the EsV-1 and mimivirus. However, the integrated sequences are more similar in structure, gene organization and GC content and at the DNA and protein level to the EsV-1 genome than to the mimiviral genome.

The genome of *Feldmannia*, another filamentous brown alga which is not able to produce virions, also contains viral fragments [32]. In contrast to *Ectocarpus*, viral DNA sequences similar to mimiviral sequences have so far not been detected in the genome of *Feldmannia *algae.

**Figure 4:**
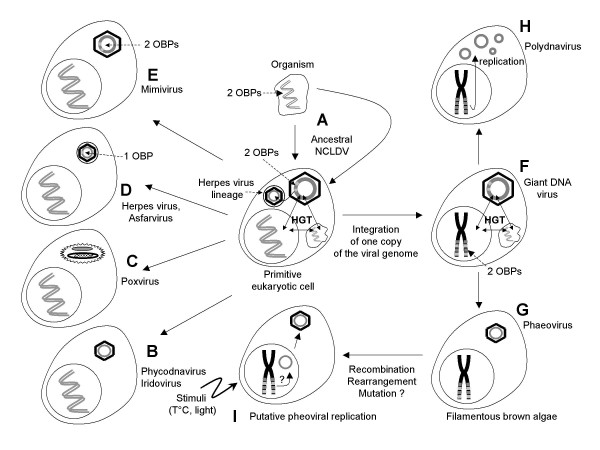
**Putative evolution of large DNA viruses**. A giant dsDNA virus or an endosymbiotic organism could be at the origin of the different classes of NCLDVs (A). This organism would have evolved mainly through genome regression as shown by the loss of the OBP ORFs (B, C, D, E). In addition, the giant microbe has probably transferred its genome to the host genome (F) and evolved to give rise to the phaeoviruses (G) and polydnaviruses (H). It is likely that horizontal gene transfer (HGT) occurred between the different organisms present in the primitive eukaryote (A, F). For example, the herpesvirus OBP might have been acquired from the NCLDV ancestor. In the case of the phaeoviruses, the viral integrated fragments could serve as templates for the production of new virions through rearrangement, recombination and mutation (I).

Therefore, the presence of the viral sequences in the genome of *Feldmannia *and *Ectocarpus *raises the question of their origin. In the case of *Ectocarpus*, because screening isolated the one copy of the viral sequences, the most obvious explanation is that these viral sequences are remnants of one ancient viral infection. We believe that these integrated sequences represent traces of an ancestral NCLDV that could be either a giant virus or an even more complex organism (Fig. 4A). The occurrence of DNA sequences similar to mimiviral sequences among the EsV-1-like fragments indicates that these sequences might come from an organism having a large genome. Such an organism was probably at least as big as the mimivirus and may even have possessed the gene content of EsV-1 and mimivirus.

Knowing that the OBP genes are either present or absent in the extant NCLDVs, the presence of intact and disrupted genes coding for the two different mimiviral-like OBPs genes allows us to propose the following scenario for the evolution of the different classes of NCLDV (Fig. 4). The different NCLDVs evolved principally through genome reduction either of a big virus or of another organism, one that had at least two OBP genes (Fig. 4). In contrast to the phycodnaviruses, the iridoviruses and poxviruses, all of which have lost both OBP genes, the asfarviruses have lost one gene each; the mimivirus, on the other hand, has conserved both genes (Fig. 4B, C, D, E). Another class of viruses, herpes viruses, might also be derived from the ancestral NCLDV because they possess one OBP and the herpes viral primase probably derived from a NCLDV primase [31]. The herpes viruses would have lost one OBP gene during the evolution (Fig. 4D). However, investigations of the structure of the capsid protein of NCLDVs and herpes viruses indicate that these viral lineages probably originated independently [33]. The herpes viruses might have therefore acquired the primase and OBP genes from a member of the NCLDV family or the NCLDV ancestor (Fig. 4A) [31,12].

In addition, the genome of the ancestral NCLDV was probably integrated into the genome of a primitive eukaryotic cell that evolved to give rise to the filamentous brown algae. During its evolution, the integrated viral genome was presumably mutated by insertion and deletion as well as disrupted by successive integrations of transposons (Fig. 1A, B). The phaeoviruses evolved principally through genome regression, which led to the loss of both OBP genes supporting the hypothesis of genome regression [25]. However, surprisingly, *Ectocarpus *algae have retained the intact OPB gene, regardless of its usefulness for algae or viruses. The product of the OBP gene is therefore probably necessary for the replication of the viral DNA. In fact, the OBP gene seems to be present only in *Ectocarpus *algae that are capable of producing viral particles and to be absent in algae in which the virus is latent (not shown).

Analysis of several NCLDVs revealed the presence of genes evolutionary related to bacterial, archaeal and eukaryotic genes [9,34]. One hypothesis suggests that horizontal gene transfer (HGT) occurred among the NCLDV ancestor, the host and endosymbionts living in the cell (Fig. 4) [13]. However, it turns out that HGT is not sufficient to account for the mosaic structure and the huge size of certain viral genomes. For example, HGT was shown to participate only somewhat in the formation of the huge genome of the mimivirus, reinforcing the idea that the ancestral NCLDV was a microbe with a large genome [14].

Another class of viruses, the polydnaviruses, could have evolved from the primitive cell that contained the ancestral NCLDV genome, given the similarities with the phaeoviruses. The polydnaviridae are a family of mutualistic dsDNA viruses associated with parasitoid wasps that parasitize other insects [35]. The genome of the polydnaviruses is present in two forms, as small DNA fragments within the genomes of wasps and as circular extra-chromosomal segments (Fig. 4H). As in EsV, the replication takes place exclusively in specialized regions of the wasp, the ovaries. The viral integrated segments are replicated, leading to the formation of the circular segments which are then injected into the prey along with the eggs. Proteins issued from the viral genes are responsible for suppressing the host's immune system and allowing the wasp's progeny to develop. In addition, although some integrated fragments are not present as circular segments after the replication, some products translated from these fragments are necessary to achieve the replication of the circular segments [36]. As mentioned above, the product of the OBP gene of region C might also be necessary for the EsV-1 genome to replicate itself. As in EsV, the transmission of the integrated segments follows Mendelian rules [37].

Even though polydnaviruses do not have any gene in common with phaeoviruses, the genome shares several characteristics with the phaeoviral genome, such as many genes that code for proteins with ankyrin domains. Furthermore, the genome of polydnavirus possesses remnants of transposon and retrovirus-like elements, such as the viral integrated fragments [38,37]. A possible symbiosis has also been mentioned for *Ectocarpus *and EsV, in part because the growth of *Ectocarpus *has been shown to be unaffected by the presence of the virus [39]. Moreover, some protein products coded by the viral genome might confer some advantages on the algae: thaumatin, for example, is a pathogen-related protein involved in plant defenses [23].

### A new system for integration, recombination and mutation?

The possible homology with polydnaviruses lets us suppose that EsV also shares a similar replication system. We propose that the EsV-1 genome originates from these viral integrated sequences by means of an original recombination and rearrangement system that is triggered by environmental stimuli (Fig. 4I). Several facts support this hypothesis. First, the brown alga *Feldmannia *sp. is able to produce viruses having genomes with two different sizes and two variants of each, depending on the temperature at which the algae are cultivated [40]. Similarly, *Feldmannia irregularis *algae can produce viruses possessing numerous genomes varying from a few kb to 180 kb [25]. In the latter case, the composition of the viral genomes and the identity of the genes sometimes differ greatly but the order of the genes remains the same. It seems unlikely that the host genome contains one copy of each viral genome. Thus, a system may exist which produces several different viral genomes using one or a few copies as template and integrated in the algal genome.

Second, the EsV-1 and FirrV-1 genomes encode a large putative integrase/ ecombinase for which no counterpart exists in current protein databases. Only the Tyr-recombinase domain at the carboxy terminus of this enzyme is similar to phage integrases (Fig. 2). Therefore, this enzyme might be involved in the integration and/or recombination of the integrated viral fragments (Fig. 4). Moreover, the presence of two tandem copies of this enzyme accompanied by an ORF encoding a transposase and relics of another one strengthens the assumption that this enzyme is also involved in transposition.

However, the discrepancy between the integrated viral sequences and EsV-1 DNA raises questions. Such a discrepancy could be explained by the intervention of a mechanism that would edit the DNA sequences by rearrangement, recombination and mutation, leading to the formation of several different genomes. For instance, the viral recombination system could resemble the systems responsible for the diversity of the immune receptor genes [41].

The difference observed in the DNA sequence could result from one of two other factors. (i) Errors caused by the DNA polymerase during the synthesis of new viral DNA [42]; or (ii) the reparation of the viral DNA during or after replication as in *E. coli*. In the bacterium, the integrity of the bacterial DNA is restored after damage when it is single-stranded [43]. This could explain the presence of single-stranded DNA regions in the EsV-1 genome [24]

It can also be argued that these integrated fragments are ancient viral sequences that are degenerating, and the EsV-1 genome is present as an extra chromosome. However, no episomal structures were observed in the reproductive cells [19]. If this turns out to be the case, it would be interesting to know whether the viral integrated fragments are replicated along with the EsV-1 genome or genetic exchange occurs between the viral genome and its integrated replica.

## Conclusion

The huge genome of certain NCLDVs is believed to be mainly due to HGT from the different hosts they might have infected during their evolution and to gene duplication. However, such an explanation is not sufficient to account, for example, for the enormous genome of the mimivirus. Here, we show that the genome of a multicellular eukaryote has acquired genes from a giant DNA virus or a unicellular organism probably as big as a mimivirus but nevertheless more similar to EsV-1 at a DNA level. A comparison with the EsV-1 and other NCLDV genomes indicates that the ancestor probably lost genes during evolution, thus corroborating the genome accretion theory. Consequently, the virus should be seen not only as a gene robber but also as a major actor in shaping the genome of brown algae; it may act to shuffle genes between species. We know that EsV-1 can infect other, related brown algae [17]. Moreover, building on former results, we propose that phaeoviruses have probably developed an original replication system. Further studies will be necessary to confirm this model.

## Methods

### Alga and virus cultures

NZVicZ14 is an infected sporophyte producing EsV-1 particles as well as zoospores depending on the cultivation conditions of the algae. The cultivation of algae as well as the isolation and purification of viruses have been previously described (Lanka et al. 1993; Kapp et al. 1997).

### DNA extraction and purification

The purification of viral DNA has been previously described (Lanka et al. 1993).

Total algal DNA was isolated from 1 g wet filaments. The algal filaments were ground to powder. The powdered filaments were mixed in 10 ml extraction buffer (0.1 M Tris, 0.7 M NaCl, 0.1 M EDTA, pH 8, 2 % (w/v) CTAB, 0.1 % SDS, 3.5 mM DETC, 1 % β-mercaptoethanol, 3% (w/v) PVPP) and shaken at room temperature for 1 hour. One volume (vol) of chloroform was then added to the cell lysate and centrifuged 10 min at 3,000 g. After centrifugation, 0.2 μg/μl RNAse A was added to the supernatant and incubated on ice for 30 min. The supernatant was extracted twice with 1 vol phenol/chloroform and 3 times with 1 vol chloroform. The DNA in the supernatant was precipitated in the presence of 0.7 vol of isopranol. The precipitated DNA was pelleted by centrifugation, dried and resuspended in 300 μl of 10 mM Tris, pH8.5.

### Genomic library

Standard methods for performing restriction digests, ligation reactions and plasmid isolations were used. A cosmid library was constructed with 50 μg algal DNA using the pWEB cosmid cloning Kit (Epigene) according to the manufacturer's instructions.

### Screening

The library was screened by colony hybridization [44]. The algal genomic library was pre-screened using a probe that consisted of EsV-1 DNA, which was partially digested with *Sau*3A. Positive clones were subjected to a second screening with the probes A and A' corresponding to the terminal regions ITRA and ITRA'. All the probes were labelled with ^32^P dCTP using a NEBlot Kit (New England Biolab). After hybridization and washing, the filters were exposed to film (Kodak XAR) for 1 to three days. Positive clones were picked and plasmid DNA was extracted and purified for further analyses.

Probes A and A' were prepared by PCR using the following primers: probe A, A5, CCGCCTTCCCACCCACATTCA and A6, GTCCAACCCATCCTCTCG; probe A', A7, TGTGGGCGAGGATGCTGTCTGAAT and A8, GTGTCGAGGCGCGTATGTTGAAAT. Thermocycling conditions were 30 cycles of denaturation at 94°C for 1 min, annealing at 60°C for 1 min, and extension at 72°C for 1 min, followed by a final 5-min extension at 72°C.

### Sequencing

A shotgun library of each positive cosmid and plasmid DNA was constructed. DNA was partially digested with *Sau*3A. The 1–5 kb DNA fragments were gel purified, ligated into the *Bam*HI site of CIP-treated plasmid pUC19 (Fermentas), and transformed into *E. coli *Max Efficiency Stbl2 (Invitrogen). DNA sequencing was carried out with the big dye kit (Perkin-Elmer) and sequences were analyzed on an ABI 377 sequencer. The shotgun sequences were assembled with the SeqManII Lasergene Software (DNASTAR Inc.) until a coverage of 10 was reached. Gaps between DNA segments were closed using PCR.

### Analysis of sequence data

The open reading frames (ORFs) were identified with the laser-gene biocomputing software package (DNASTAR Inc.). Homology searches were carried out with the blast program [45]. Protein motifs were searched against the SMART [46] and Pfam [47] databases. Sequence alignments were performed with the Megalign program (DNASTAR Inc.).

### Nucleotide sequence accession numbers

The DNA nucleotide sequences of the contigs have been deposited in GenBank under the following accession numbers: EU254745, EU254746 and EU254747

## Authors' contributions

ND conceived of the study, carried out the molecular genetic studies, provided the biological interpretation of the results, and finalized the manuscript. WB drafted the manuscript. All authors read and approved the final manuscript.

## References

[B1] Villarreal LP DeFilippis VR A hypothesis for DNA viruses as the origin of eukaryotic replication proteins J Virol 2000 74 15 7079 7084 10.1128/JVI.74.15.7079-7084.2000 10888648 PMC112226

[B2] Bell PJ Viral eukaryogenesis: was the ancestor of the nucleus a complex DNA virus? J Mol Evol 2001 53 3 251 256 10.1007/s002390010215 11523012

[B3] Bell PJ Sex and the eukaryotic cell cycle is consistent with a viral ancestry for the eukaryotic nucleus J Theor Biol 2006 10.1016/j.jtbi.2006.05.015 16846615

[B4] Takemura M Poxviruses and the origin of the eukaryotic nucleus J Mol Evol 2001 52 5 419 425 11443345 10.1007/s002390010171

[B5] Filee J Forterre P Sen-Lin T Laurent J Evolution of DNA polymerase families: evidences for multiple gene exchange between cellular and viral proteins J Mol Evol 2002 54 6 763 773 10.1007/s00239-001-0078-x 12029358

[B6] Claverie JM Viruses take center stage in cellular evolution Genome Biol 2006 7 6 110 10.1186/gb-2006-7-6-110 16787527 PMC1779534

[B7] Forterre P The origin of viruses and their possible roles in major evolutionary transitions Virus Research 2006 117 1 5 16 10.1016/j.virusres.2006.01.010 16476498

[B8] Van Etten JL Graves MV Muller DG Boland W Delaroque N Phycodnaviridae--large DNA algal viruses Arch Virol 2002 147 8 1479 1516 10.1007/s00705-002-0822-6 12181671

[B9] Raoult D Audic S Robert C Abergel C Renesto P Ogata H La Scola B Suzan M Claverie JM The 1.2-megabase genome sequence of mimivirus Science 2004 306 5700 1344 1350 10.1126/science.1101485 15486256

[B10] Wilson WH Schroeder DC Allen MJ Holden MT Parkhill J Barrell BG Churcher C Hamlin N Mungall K Norbertczak H Quail MA Price C Rabbinowitsch E Walker D Craigon M Roy D Ghazal P Complete genome sequence and lytic phase transcription profile of a Coccolithovirus Science 2005 309 5737 1090 1092 10.1126/science.1113109 16099989

[B11] Iyer LM Aravind L Koonin EV Common origin of four diverse families of large eukaryotic DNA viruses J Virol 2001 75 23 11720 11734 10.1128/JVI.75.23.11720-11734.2001 11689653 PMC114758

[B12] Iyer LM Balaji S Koonin EV Aravind L Evolutionary genomics of nucleo-cytoplasmic large DNA viruses Virus Res 2006 117 1 156 184 10.1016/j.virusres.2006.01.009 16494962

[B13] Koonin EV Senkevich TG Dolja VV The ancient Virus World and evolution of cells Biol Direct 2006 1 29 29 10.1186/1745-6150-1-29 16984643 PMC1594570

[B14] Moreira D López-García P Comment on "The 1.2-megabase genome sequence of Mimivirus" Science 2005 308 5725 1114 10.1126/science.1110820 15905382

[B15] Suhre K Audic S Claverie JM Mimivirus gene promoters exhibit an unprecedented conservation among all eukaryotes Proceedings of the National Academy of Sciences of the United States of America 2005 102 41 14689 14693 10.1073/pnas.0506465102 16203998 PMC1239944

[B16] Suhre K Gene and genome duplication in Acanthamoeba polyphaga Mimivirus Journal of Virology 2005 79 22 14095 14101 10.1128/JVI.79.22.14095-14101.2005 16254344 PMC1280231

[B17] Muller DG Kapp M Knippers R Viruses in marine brown algae Adv Virus Res 1998 50 49 67 9520996 10.1016/s0065-3527(08)60805-2

[B18] Brautigam M Klein M Knippers R Muller DG Inheritance and meiotic elimination of a virus genome in the host ectocarpus siliculosus (phaeophyceae) Journal of Phycology 1995 31 5 823 827 10.1111/j.0022-3646.1995.00823.x

[B19] Delaroque N Maier I Knippers R Muller DG Persistent virus integration into the genome of its algal host, Ectocarpus siliculosus (Phaeophyceae) Journal of General Virology 1999 80 Pt 6 1367 1370 10374952 10.1099/0022-1317-80-6-1367

[B20] Mueller DG Mendelian segregation of a virus genome during host meiosis in the marine brown alga Ectocarpus siliculosus J Plant Physiol 1991 137 739 743

[B21] Müller DGK H. Stache, B. Lanka, S. A virus infection in the marine brown alga Ectocarpus siliculosus (Phaeophyceae) Botanica Acta 1990 103 72 82

[B22] Lanka ST Klein M Ramsperger U Muller DG Knippers R Genome structure of a virus infecting the marine brown alga Ectocarpus siliculosus Virology 1993 193 2 802 811 10.1006/viro.1993.1189 8460486

[B23] Delaroque N Muller DG Bothe G Pohl T Knippers R Boland W The complete DNA sequence of the Ectocarpus siliculosus virus EsV-1 genome Virology 2001 287 1 112 132 10.1006/viro.2001.1028 11504547

[B24] Klein M Lanka S Muller D Knippers R Single-stranded regions in the genome of the Ectocarpus siliculosus virus Virology 1994 202 2 1076 1078 10.1006/viro.1994.1443 8030215

[B25] Delaroque N Boland W Muller DG Knippers R Comparisons of two large phaeoviral genomes and evolutionary implications J Mol Evol 2003 57 6 613 622 10.1007/s00239-003-2501-y 14745530

[B26] Muller DG Sengco M Wolf S Brautigam M Schmid CE Kapp M Knippers R Comparison of two DNA viruses infecting the marine brown algae Ectocarpus siliculosus and E. fasciculatus J Gen Virol 1996 77 ( Pt 9) 2329 2333 8811034 10.1099/0022-1317-77-9-2329

[B27] Deneke J Burgin AB Wilson SL Chaconas G Catalytic residues of the telomere resolvase ResT: a pattern similar to, but distinct from, tyrosine recombinases and type IB topoisomerases J Biol Chem 2004 279 51 53699 706. Epub 2004 Oct 6. 10.1074/jbc.M409001200 15471873

[B28] Nunes-Duby SE Kwon HJ Tirumalai RS Ellenberger T Landy A Similarities and differences among 105 members of the Int family of site-specific recombinases Nucleic Acids Res 1998 26 2 391 406 10.1093/nar/26.2.391 9421491 PMC147275

[B29] O'Day DH Suhre K Myre MA Chatterjee-Chakraborty M Chavez SE Isolation, characterization, and bioinformatic analysis of calmodulin-binding protein cmbB reveals a novel tandem IP22 repeat common to many Dictyostelium and Mimivirus proteins Biochem Biophys Res Commun 2006 346 3 879 888 10.1016/j.bbrc.2006.05.204 16777069

[B30] Lehman IR Boehmer PE Replication of herpes simplex virus DNA J Biol Chem 1999 274 40 28059 28062 10.1074/jbc.274.40.28059 10497152

[B31] Iyer LM Koonin EV Leipe DD Aravind L Origin and evolution of the archaeo-eukaryotic primase superfamily and related palm-domain proteins: structural insights and new members Nucleic Acids Research 2005 33 12 3875 3896 10.1093/nar/gki702 16027112 PMC1176014

[B32] Lee AM Ivey RG Meints RH Repetitive DNA insertion in a protein kinase ORF of a latent FSV (Feldmannia sp. virus) genome Virology 1998 248 1 35 45 10.1006/viro.1998.9245 9705253

[B33] Bamford DH Grimes JM Stuart DI What does structure tell us about virus evolution? Curr Opin Struct Biol 2005 15 6 655 63. Epub 2005 Nov 3. 10.1016/j.sbi.2005.10.012 16271469

[B34] Dunigan DD Fitzgerald LA Van Etten JL Phycodnaviruses: A peek at genetic diversity Virus Research 2006 117 1 119 132 10.1016/j.virusres.2006.01.024 16516998

[B35] Kroemer JA Webb BA Polydnavirus genes and genomes: emerging gene families and new insights into polydnavirus replication Annu Rev Entomol 2004 49 431 456 10.1146/annurev.ento.49.072103.120132 14651471

[B36] Deng L Stoltz DB Webb BA A gene encoding a polydnavirus structural polypeptide is not encapsidated Virology 2000 269 2 440 450 10.1006/viro.2000.0248 10753722

[B37] Webb BA Strand MR Dickey SE Beck MH Hilgarth RS Barney WE Kadash K Kroemer JA Lindstrom KG Rattanadechakul W Shelby KS Thoetkiattikul H Turnbull MW Witherell RA Polydnavirus genomes reflect their dual roles as mutualists and pathogens Virology 2006 347 1 160 174 10.1016/j.virol.2005.11.010 16380146

[B38] Espagne E Dupuy C Huguet E Cattolico L Provost B Martins N Poirie M Periquet G Drezen JM Genome sequence of a polydnavirus: insights into symbiotic virus evolution Science 2004 306 5694 286 289 10.1126/science.1103066 15472078

[B39] Campo ED Ramazanov Z Garcia-Reina G Müller DG Photosynthetic responses and growth performance of virus-infected and noninfected Ectocarpus siliculosus Phycologia 1997 36 3 186 189

[B40] Ivey RG Henry EC Lee AM Klepper L Krueger SK Meints RH A Feldmannia algal virus has two genome size-classes Virology 1996 220 2 267 273 10.1006/viro.1996.0314 8661377

[B41] Litman GW Cannon JP Dishaw LJ Reconstructing immune phylogeny: new perspectives Nat Rev Immunol 2005 5 11 866 879 10.1038/nri1712 16261174 PMC3683834

[B42] Speyer JF Mutagenic DNA polymerase Biochem Biophys Res Commun 1965 21 1 6 8 10.1016/0006-291X(65)90417-1 5865492

[B43] Viswanathan M Lovett ST Single-strand DNA-specific exonucleases in Escherichia coli. Roles in repair and mutation avoidance Genetics 1998 149 1 7 16 9584082 10.1093/genetics/149.1.7 PMC1460129

[B44] Sambrook J Fritsch EF Maniatis T Molecular Cloning - A Laboratory Manual 1989 2nd New York, Cold Spring Harbor Press, Cold Spring Harbor

[B45] Altschul SF Gish W Miller W Myers EW Lipman DJ Basic local alignment search tool J Mol Biol 1990 215 3 403 410 2231712 10.1016/S0022-2836(05)80360-2

[B46] Schultz J Milpetz F Bork P Ponting CP SMART, a simple modular architecture research tool: identification of signaling domains Proc Natl Acad Sci U S A 1998 95 11 5857 5864 10.1073/pnas.95.11.5857 9600884 PMC34487

[B47] Finn RD Mistry J Schuster-Bockler B Griffiths-Jones S Hollich V Lassmann T Moxon S Marshall M Khanna A Durbin R Eddy SR Sonnhammer EL Bateman A Pfam: clans, web tools and services Nucleic Acids Res 2006 34 Database issue D247 51 10.1093/nar/gkj149 16381856 PMC1347511

